# Structures of full-length VanR from *Streptomyces coelicolor* in both the inactive and activated states

**DOI:** 10.1107/S2059798321006288

**Published:** 2021-07-29

**Authors:** Lina J. Maciunas, Nadia Porter, Paula J. Lee, Kushol Gupta, Patrick J. Loll

**Affiliations:** a Department of Biochemistry and Molecular Biology, Drexel University College of Medicine, Philadelphia, PA 19102, USA; b Graduate Program in Biochemistry, Drexel University College of Medicine, Philadelphia, PA 19102, USA; c Summer Undergraduate Research Fellowship Program, Drexel University College of Medicine, Philadelphia, PA 19102, USA; d Department of Biochemistry and Biophysics, University of Pennsylvania Perelman School of Medicine, Philadelphia, PA 19104, USA

**Keywords:** vancomycin resistance, X-ray crystallography, two-component systems, response regulators, *Streptomyces coelicolor*, full-length VanR

## Abstract

Vancomycin has historically been used as a last-resort treatment for serious bacterial infections. However, vancomycin resistance has become widespread in certain pathogens, presenting a serious threat to public health. Resistance to vancomycin is conferred by a suite of resistance genes, the expression of which is controlled by the VanR–VanS two-component system. VanR is the response regulator in this system; in the presence of vancomycin, VanR accepts a phosphoryl group from VanS, thereby activating VanR as a transcription factor and inducing expression of the resistance genes. This paper presents the X-ray crystal structures of full-length VanR from *Streptomyces coelicolor* in both the inactive and activated states at resolutions of 2.3 and 2.0 Å, respectively. Comparison of the two structures illustrates that phosphorylation of VanR is accompanied by a disorder-to-order transition of helix 4, which lies within the receiver domain of the protein. This transition generates an interface that promotes dimerization of the receiver domain; dimerization in solution was verified using analytical ultracentrifugation. The inactive conformation of the protein does not appear intrinsically unable to bind DNA; rather, it is proposed that in the activated form DNA binding is enhanced by an avidity effect contributed by the receiver-domain dimerization.

## Abstract 2

Crystal structures are presented of the full-length VanR protein in both the inactive and activated states. Activation involves a disorder-to-order transition in a critical helix, which creates an interface for dimerization.

## 1. Introduction   

Two-component systems are critical to the survival of bacteria, mediating processes that include cell division, chemotaxis and antibiotic resistance. Each two-component system is composed of a sensor histidine kinase and a response regulator, which together coordinate an appropriate cellular response to an environmental stimulus. The kinase detects the stimulus and, in response, autophosphorylates on a histidine residue. This phosphoryl group is then transferred to an aspartate residue on the response regulator, thereby activating it (West & Stock, 2001 ▸). Once activated, the response regulator produces an output that represents a suitable reaction to the stimulus. Depending on the response regulator, this output can involve binding to DNA, interacting with another protein, or expressing an enzymatic activity (Galperin, 2006 ▸).

VanR is the response regulator of the VanR–VanS two-component system, which controls the expression of a vancomycin-resistant phenotype in many bacteria, including soil microbes such as *Streptomyces coelicolor* as well as serious human pathogens such as vancomycin-resistant enterococci (VRE; Hong *et al.*, 2008 ▸). VanR is composed of an N-terminal receiver domain, which contains the conserved aspartate that is the target for phosphorylation, and a C-terminal DNA-binding domain, which enables VanR to function as a transcription factor. Upon phosphorylation, VanR is thought to dimerize, bind to promoters within the vancomycin-resistance operon, and activate transcription of the resistance genes (Arthur *et al.*, 1992 ▸; Holman *et al.*, 1994 ▸; Evers & Courvalin, 1996 ▸; Depardieu *et al.*, 2005 ▸). However, the structural transitions associated with VanR activation remain incompletely understood.

VanR belongs to the OmpR/PhoB class of response regulators (Nguyen *et al.*, 2015 ▸). This class of proteins has been the focus of extensive structural characterization, and structures are available of receiver domains in their inactive and activated states (Bachhawat *et al.*, 2005 ▸; Toro-Roman, Mack *et al.*, 2005 ▸; Toro-Roman, Wu *et al.*, 2005 ▸), DNA-binding domains in their free and DNA-bound states (Blanco *et al.*, 2002 ▸; Wang *et al.*, 2007 ▸; He *et al.*, 2016 ▸) and full-length proteins in either inactive or activated states (Buckler *et al.*, 2002 ▸; Robinson *et al.*, 2003 ▸; Nowak *et al.*, 2006 ▸; Friedland *et al.*, 2007 ▸; Narayanan *et al.*, 2014 ▸; Lou *et al.*, 2015 ▸). However, to the best of our knowledge, there are no full-length proteins in the OmpR/PhoB class for which structures are known of both the inactive and activated forms. Indeed, only a few response regulators of any class have been crystallized in both forms, limiting our understanding of the mechanistic basis of activation.

In this paper, we present the structures of full-length VanR from *Streptomyces coelicolor* (VanR_Sc_) in its inactive and activated states. These represent the first reported structures of any VanR protein, as well as the first report of structures of an OmpR/PhoB-type response regulator in both activity states. Comparison of the two VanR_Sc_ structures suggests that dimerization is the primary mechanism underlying activation.

## 2. Materials and methods   

### 2.1. Cloning, expression and purification   

The gene for VanR_Sc_ (NC_003888.3) was amplified from the genome of *S. coelicolor* M145 (ATCC BAA-471; Bentley *et al.*, 2002 ▸) using forward primer 5′-AGA TTG GTG GCG GAA TGC GTG TGC TGA TTG TCG AG-3′ and reverse primer 5′-GAG GAG AGT TTA GAC ATT ACT ATC CAC CGT CGC CGC C-3′. The amplified gene was subcloned into the in-house vector pETHSUL (Weeks *et al.*, 2007 ▸), generating an expression construct for VanR_Sc_ containing an N-terminal His_6_-SUMO tag that could be cleaved with SUMO hydrolase. To facilitate SUMO cleavage, a glycine residue was inserted directly upstream of the first residue of VanR_Sc_. Thus, the final purified VanR protein consists of residues 1–231 plus the additional N-terminal glycine. The VanR_Sc_-SUMO fusion protein was expressed in *Escherichia coli* BL21(DE3) cells, which were grown in 2.5 l Ultra Yield flasks (Thomson Instrument Co., part No. 931136-B) in lysogeny broth (LB) with shaking at 225 rev min^−1^. Cultures were grown to mid-exponential phase at 37°C, after which the temperature was reduced to 18°C and protein expression was induced with 0.2 m*M* isopropyl β-d-1-thiogalactopyranoside (IPTG) for 18 h. The cells were harvested by centrifugation and the pellets were stored at −80°C until further use.

Pelleted cells from 4 l of growth culture were thawed and resuspended in IMAC buffer *A* (300 m*M* NaCl, 40 m*M* Tris pH 8, 25 m*M* imidazole) containing 10 µg ml^−1^ DNase, 2 µg ml^−1^ RNase, 1 m*M* MgCl_2_ and Pierce EDTA-free protease-inhibitor tablets. The resuspended cells were lysed in a C5 Emulsiflex cell homogenizer (Avestin, Ottawa, Ontario, Canada) at 103–137 MPa. The lysate was clarified by centrifuging the sample at 208 000*g* for 1 h. The resultant supernatant was syringe-filtered through a sterile 0.45 µm filter and was then loaded onto a 5 ml HiTrap IMAC-HP column (GE Healthcare) equilibrated in IMAC buffer *A*. The column was washed with 25 ml IMAC buffer *A* followed by 25 ml 10% IMAC buffer *B* (300 m*M* NaCl, 40 m*M* Tris pH 8, 350 m*M* imidazole). The protein was eluted with a 25 ml gradient from 10% to 100% IMAC buffer *B*. Fractions containing the fusion protein were pooled and the recombinant yeast SUMO hydrolase dtUD1 (Weeks *et al.*, 2007 ▸) was added to the sample at a final concentration of 5 µg ml^−1^. The sample was dialyzed overnight against IMAC buffer *A* lacking imidazole using 3.5 kDa molecular-weight cutoff SnakeSkin dialysis tubing (Thermo Fisher Scientific, catalog No. 88244). The dialysate was passed over the IMAC column re-equilibrated in IMAC buffer *A* to capture the His-SUMO fusion partner. The flowthrough containing VanR_Sc_ was collected, concentrated and injected onto a Sephacryl S200 size-exclusion column (GE Healthcare) equilibrated in IMAC buffer *A* lacking imidazole. The peak fractions were collected, concentrated and filtered through a 0.22 µm filter. The concentration was determined from the *A*_280_ using the calculated extinction coefficient ɛ = 14 440 *M* ^−1^ cm^−1^.

### 2.2. Analytical ultracentrifugation   

Sedimentation-velocity analytical ultracentrifugation experiments were performed at 20°C with an XL-A analytical ultracentrifuge (Beckman-Coulter, Brea, California, USA) and a TiAn60 rotor with two-channel charcoal-filled Epon centerpieces and quartz windows. The VanR_Sc_ protein was dissolved in 50 m*M* Tris pH 7.5, 50 m*M* NaCl, 10 m*M* MgCl_2_ with or without 5 m*M* BeSO_4_ and 35 m*M* NaF. Data were collected with detection at 280 nm. Complete sedimentation-velocity profiles were recorded every 30 s at 40 000 rev min^−1^. Data were fitted using the *c*(*s*) or *c*(*s*, *f*/*f*_0_) distribution implementations of the Lamm equation as implemented in *SEDFIT* (Schuck, 2000 ▸) and corrected for *S*_20,w_. Direct fitting of association models was performed using *SEDPHAT* (Vistica *et al.*, 2004 ▸).

Sedimentation-equilibrium analytical ultracentrifugation data were collected at 4°C with detection at 280 nm and a TiAn60 rotor with six-channel charcoal-filled Epon centerpieces and quartz windows at three sample concentrations at 18 000, 20 000 and 22 000 rev min^−1^. Analyses were carried out using global fits to data acquired at multiple speeds at four concentrations with strict mass conservation using* SEDPHAT* (Vistica *et al.*, 2004 ▸). Error estimates for equilibrium constants and fit masses were determined from a 1000-iteration Monte Carlo simulation.

The partial specific volume ( 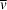 ), solvent density (ρ) and viscosity (η) were derived from the chemical composition by *SEDNTERP* (Laue *et al.*, 1992 ▸). Figures were created using *GUSSI* (Brautigam, 2015 ▸).

### 2.3. Crystallization and X-ray data collection   

The inactive state of VanR_Sc_ was crystallized by microbatch under Al’s Oil (Hampton Research, catalog No. HR3-413; Chayen *et al.*, 1992 ▸; D’Arcy *et al.*, 1996 ▸). Freshly purified protein was dialyzed against 500 m*M* NaCl, 5 m*M* MgCl_2_, 10%(*v*/*v*) glycerol, 40 m*M* Tris pH 8 and concentrated to ∼11.3 mg ml^−1^. The protein solution (0.5 µl) was combined with an equal volume of crystallization condition C1 from Rigaku’s Wizard Classic 1 and 2 screens [0.2 *M* MgCl_2_, 0.1 *M* Tris pH 8.5, 30%(*w*/*v*) PEG 400] using an Oryx6 Robot (Douglas Instruments, Berkshire, UK). Crystallization trays were incubated at 4°C and thin needle-like crystals appeared within three weeks. Crystals were harvested without additional cryoprotectant and were flash-cooled by plunging into liquid nitrogen.

Crystals of the activated state of VanR_Sc_ were prepared by co-crystallizing freshly prepared VanR_Sc_ with beryllium fluoride ( 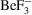 ) by microbatch under oil. The protein (in IMAC buffer *A* lacking imidazole) was concentrated to 11.3 mg ml^−1^. A 10× 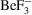 stock solution was prepared consisting of 50 m*M* BeSO_4_, 350 m*M* NaF, 70 m*M* MgCl_2_. The 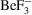 stock was combined with the protein in a 1:10(*v*:*v*) ratio, resulting in a final protein concentration of ∼10.2 mg ml^−1^. 0.5 µl of the VanR_Sc_– 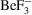 mixture was combined with 0.5 µl crystallization condition F5 from Rigaku’s Wizard Classic 1 and 2 screens (2.5 *M* NaCl, 0.1 *M* Tris pH 7, 0.2 *M* MgCl_2_). The crystallization tray was incubated at room temperature and rod-like crystals grew after three days. The crystals were dragged through the oil as a cryoprotectant and then plunged into liquid nitrogen.

Diffraction data for both crystal forms were measured on the AMX beamline of the National Synchrotron Light Source II (NSLS-II). Data-collection details are summarized in Table 1 ▸.

### 2.4. Structure determination and refinement   

The data collected from crystals of VanR_Sc_ in both activity states were processed by *XDS* and scaled with *XSCALE* (Kabsch, 2010 ▸). The structure of the activated VanR_Sc_ was determined by molecular replacement with *Phaser* in *Phenix* (Zwart *et al.*, 2008 ▸; Liebschner *et al.*, 2019 ▸) utilizing two probes. The probes were chosen using *BLAST* searches employing the sequences of the VanR_Sc_ receiver and DNA-binding domains; residues 19–119 from PDB entry 5uic (Milton *et al.*, 2017 ▸) were used to spatially orient the receiver domain and residues 134–220 from PDB entry 1kgs (Buckler *et al.*, 2002 ▸) were used to orient the DNA-binding domain. The structure of the inactive VanR_Sc_ was determined by molecular replacement using the two domains of the activated protein as probes. Models of the inactive VanR_Sc_ and activated VanR_Sc_ were built using the *AutoBuild* function in *Phenix*. These models were iteratively adjusted in *Coot* (Emsley *et al.*, 2010 ▸) and refined in *Phenix*. The quality of the final models was assessed by both *MolProbity* (Chen *et al.*, 2010 ▸) and the *R*_free_ value. The *R*_free_ value was based on 5% of the total reflections chosen at random prior to refinement. The final refinement statistics are shown in Table 1 ▸. Coordinates and structure factors were deposited with the Protein Data Bank (PDB entries 7lz9 and 7lza for the inactive and activated proteins, respectively). Raw diffraction images are available from the Zenodo repository (https://www.zenodo.org) using the following digital object identifiers: https://doi.org/10.5281/zenodo.4594513 for inactive VanR_Sc_ and https://doi.org/10.5281/zenodo.4593691 for activated VanR_Sc_.

All figures were made using *PyMOL* (version 2.3; Schrödinger). Interdomain interfaces were identified using both *AREAIMOL* within *CCP*4 version 7.0 (Lee & Richards, 1971 ▸; Saff & Kuijlaars, 1997 ▸; Winn *et al.*, 2011 ▸) and the *PISA* web server (Krissinel & Henrick, 2007 ▸). *TM-align* was used for the superposition of analogous domains (Zhang & Skolnick, 2005 ▸). *HELANAL-Plus* was used to calculate helix axes (Kumar & Bansal, 2012 ▸).

## 3. Results   

### 3.1. Structure determination   

**Figure 1:**
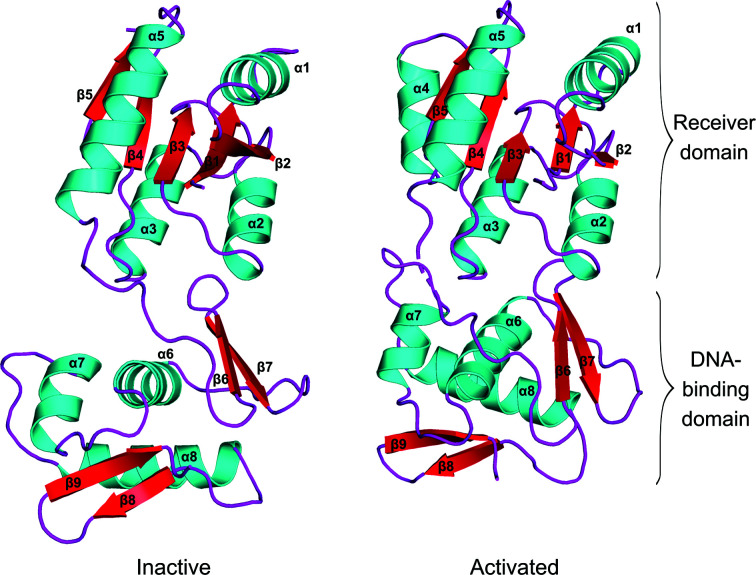
Crystal structures of full-length VanR_Sc_ in the inactive and activated states. The receiver and DNA-binding domains are indicated. Secondary structures are colored as follows: β-strands, red; α-­helices, cyan; loops, magenta. Numbering is shown for α-helices and β-strands. Helix α4 is absent from the inactive VanR_Sc_ structure, reflecting presumptive disorder. The loop connecting the receiver and DNA-binding domains is fully ordered in the inactive state, but is disordered at His121 in the activated state (indicated by dashes in the right-hand structure). Stereo versions of both panels can be found in Supplementary Fig. S1.

Full-length recombinant VanR_Sc_ was produced in *E. coli* and purified by subtractive immobilized metal-ion chromatography and gel filtration. The activated state was generated by treating the protein with beryllium fluoride, which has proven to act as a faithful mimic of aspartate phosphorylation in a variety of response regulators (Wemmer & Kern, 2005 ▸). X-ray crystal structures were determined for the inactive and activated states at resolutions of 2.3 and 2.0 Å, respectively. Both activity states crystallized in the same *P*6_5_22 space group but with different unit-cell dimensions; in both crystal forms the asymmetric unit contained a monomer (Fig. 1 ▸). For both conformational states, no electron density was observed for the 12 C-terminal residues (221–232), suggesting a highly flexible C-terminal tail. Details of the structure determination and refinement are given in Table 1 ▸.

### 3.2. Overall description of the structures   

The receiver domains of many response regulators have been crystallized in the inactive and activated states, and the receiver domain of VanR_Sc_ is very similar to those seen previously, with root-mean-square deviations (r.m.s.d.s) for C^α^-atom positions ranging from 1.1 to 2.0 Å (Supplementary Table S1; Stock *et al.*, 1989 ▸; Robinson *et al.*, 2000 ▸). The domain adopts an α/β-sandwich fold composed of a central five-stranded parallel β-sheet with a 2–1–3–4–5 topology surrounded by three α-helices on one side (α2, α3 and α4) and two α-helices on the other (α1 and α5).

As is true for other members of the OmpR/PhoB class of response regulators, the DNA-binding domain of VanR_Sc_ adopts a winged helix–turn–helix motif composed of three α-helices (α6, α7 and α8) followed by a C-terminal β-hairpin (Martínez-Hackert & Stock, 1997 ▸). α8 is also referred to as the recognition helix, and is expected to bind within the major groove of DNA, making specific contacts with bases, sugars and the phosphate backbone of DNA. In addition to the winged-helix motif, DNA-binding domains of the OmpR/PhoB class typically contain a four-stranded antiparallel β-sheet upstream of the winged helix–turn–helix. In VanR_Sc_, this β-sheet contains only two strands, along with the possible vestige of a third (Fig. 1 ▸). The lack of a full four-stranded β-sheet is uncommon but not unprecedented; for example, the DNA-binding domain of PmrA contains a sheet with only three antiparallel β-strands (Lou *et al.*, 2015 ▸). In spite of the difference in this sheet region, the DNA-binding domain of VanR_Sc_ is very similar overall to those of other OmpR/PhoB response regulators, with r.m.s.d.s for C^α^ atoms ranging from 1.3 to 2.7 Å (Supplementary Table S2).

In OmpR/PhoB response regulators, the length of the linker connecting the receiver and DNA-binding domains ranges from five to 21 amino acids (Martínez-Hackert & Stock, 1997 ▸). In VanR_Sc_ the linker contains 11 residues; in addition, in the activated state, partial unwinding of the C-terminus of α5 extends the linker length by three residues. The functional significance of this length difference is unclear, but it may provide additional flexibility that allows optimal positioning of the DNA-binding domains upon their DNA target. While one might expect long linkers such as those in the VanR_Sc_ structures to be highly flexible, the linkers are well ordered in the crystals of both activity states. In the structure of inactive VanR_Sc_ the linkers from two adjacent molecules in the crystal lattice associate in an antiparallel manner, leading to the formation of a symmetric pair of hydrogen bonds between the carbonyl O atom of Arg123 and the amide proton of Arg123′ of the symmetry mate, along with a similarly symmetric pair of hydrogen bonds between the side chain of Arg123 and the carbonyl O atom of Pro124′. In the activated VanR_Sc_ structure the linker does not participate in any crystal contacts and yet remains almost completely ordered, with the exception of a single chain break at His121.

### 3.3. Comparison of the receiver-domain structure with those of other response regulators   

**Figure 2:**
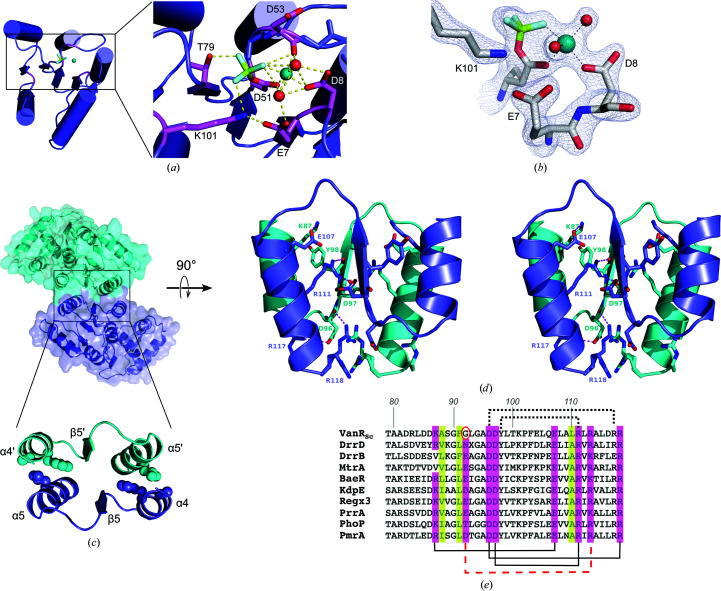
Activation of VanR_Sc_. (*a*) A view from above the site of phosphorylation. The phosphoryl acceptor Asp51 lies at the end of the third β-strand and is shown bound to the beryllium fluoride phosphomimetic. The F atoms (pale blue) interact with the side chains of Thr79 and Lys101 and the backbone carbonyl of Asp53. A magnesium ion (shown in teal) is also present at the phosphorylation site, and is octahedrally coordinated by the side chains of Asp8, Glu7 and Asp51, the backbone carbonyl of Asp53 and two water molecules (shown as red spheres). (*b*) 2*F*_o_ − *F*_c_ electron-density map showing the site of phosphorylation. A σ_A_-weighted map (Read, 1986 ▸) contoured at 1.6σ is shown. A stereo version of this panel can be found in Supplementary Fig. S2. (*c*) The activated dimer, shown in both surface and cartoon representation. One protomer is colored cyan and the other is colored slate blue. The boxed region highlights the dimer interface that forms around α4–β5–α5. Hydrophobic contacts along the outer edges of the dimer interface stabilize dimer formation; the amino-acid side chains responsible for these contacts are shown as spheres. (*d*) Stereoview of polar contacts stabilizing the core of the dimer interface. The orientation shown is rotated 90° about a horizontal axis relative to the orientation shown in (*c*). (*e*) Conservation of dimer-interface residues for OmpR/PhoB response regulators. Hydrophobic residues are highlighted in lime green and polar residues are highlighted in pink. The brackets below the sequences indicate the typical salt bridges formed within the interface, while the brackets above indicate interactions that are specific to VanR_Sc_. Because VanR_Sc_ contains a glycine at position 92 (circled), it does not form the typical 92–113 interaction (represented by the red dashed line); instead, an alternative hydrogen-bond interaction is formed between Tyr98 and Arg111. Numbering for the VanR_Sc_ sequence is shown at the top. For convenience, the brackets representing interactions are drawn connecting two residues within a single stretch of sequence; however, the actual interactions occur between two different protomers, across the dimer interface.

The active site of the activated form of VanR_Sc_ resembles those found in other activated receiver-domain structures (Yan *et al.*, 1999 ▸). The active site centers around the highly conserved receiver of the phosphoryl group, Asp51, which is located at the end of β3. Similar to what is seen in other activated receiver-domain structures, the 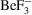 ion is coordinated by Asp51, together with a magnesium ion, to form a phosphomimetic (Fig. 2 ▸ *a* and 2 ▸ *b*). The Be atom is bound to one of the carboxylate O atoms of Asp51, while the F atoms interact with the side chains of Thr79 and Lys101 and with the magnesium ion. The magnesium displays octahedral coordination, with its ligands including an F atom, the side chains of Asp51 and Asp8, the backbone carbonyl O atom of Asp53 and two water molecules. These water molecules interact with each other as well as with the side chains of Asp51, Glu7, Asp8 and Lys101, making them integral components in a network of hydrogen bonds that spans the active site.

Once activated, the receiver domains of the OmpR/PhoB family assemble into twofold-symmetric dimers, with the α4–β5–α5 surface forming the dimer interface (Toro-Roman, Wu *et al.*, 2005 ▸). For activated VanR_Sc_, the crystal asymmetric unit only contains a monomer, but a dimer with the expected interface is formed by crystal symmetry (Fig. 2 ▸ *c*). The α4–β5–α5 interface buries 846 Å^2^ of surface area, with the fraction of atoms completely buried (*f*_BU_) equal to 0.34; both of these values are consistent with this interface being biologically relevant (Ponstingl *et al.*, 2000 ▸; Janin *et al.*, 2007 ▸). In contrast, in the crystals of the inactive form of VanR_Sc_ none of the lattice contacts appear to correspond to biologically meaningful interfaces.

The α4–β5–α5 dimer is specific to the OmpR/PhoB class of response regulators, and relies on a set of conserved inter­actions that are unique to this class (Toro-Roman, Mack *et al.*, 2005 ▸). These include hydrophobic interactions formed at the outer edges of the contact surface and polar interactions that stabilize the core of the interface. In VanR_Sc_ the hydrophobic interactions involve Ala88 and Phe91, which lie on α4 of one protomer, and Leu110, which is located on α5 of the facing protomer (Fig. 2 ▸ *c*). The polar interactions involve five buried contacts across the dimer interface, which are made between the following pairs of residues: Glu107/Lys87, Asp97/Arg111, Asp96 (main chain)/Arg118, Asp96 (side chain)/Arg117 and Tyr98/Arg111 (Fig. 2 ▸ *d*). Interactions involving the first three of these interacting pairs are highly conserved in OmpR/PhoB response regulators, while interactions involving the fourth are seen in some but not all members of the family (Fig. 2 ▸ *e*). The fifth interaction (Tyr98–Arg111) is atypical and appears to replace an interaction found in most family members, but not in VanR_Sc_. Normally, OmpR/PhoB response regulators contain a salt bridge between a glutamate at the C-terminus of α4 and an arginine in α5; in VanR_Sc_ the corresponding residues are 92 and 113. However, VanR_Sc_ has a glycine at residue 92, rather than a glutamate, and thus instead of the typical Glu–Arg salt bridge it forms an alternative polar contact between the backbone carbonyl of Tyr98 and the side chain of Arg111 (Fig. 2 ▸ *e*). Despite this difference, the overall pattern of interactions within the dimer interface and the active site closely corresponds to those found in other activated response regulators, supporting the assumption that the VanR_Sc_– 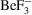 complex closely mimics the conformation of the phosphorylated protein.

### 3.4. Experimental confirmation of oligomer formation   

**Figure 3:**
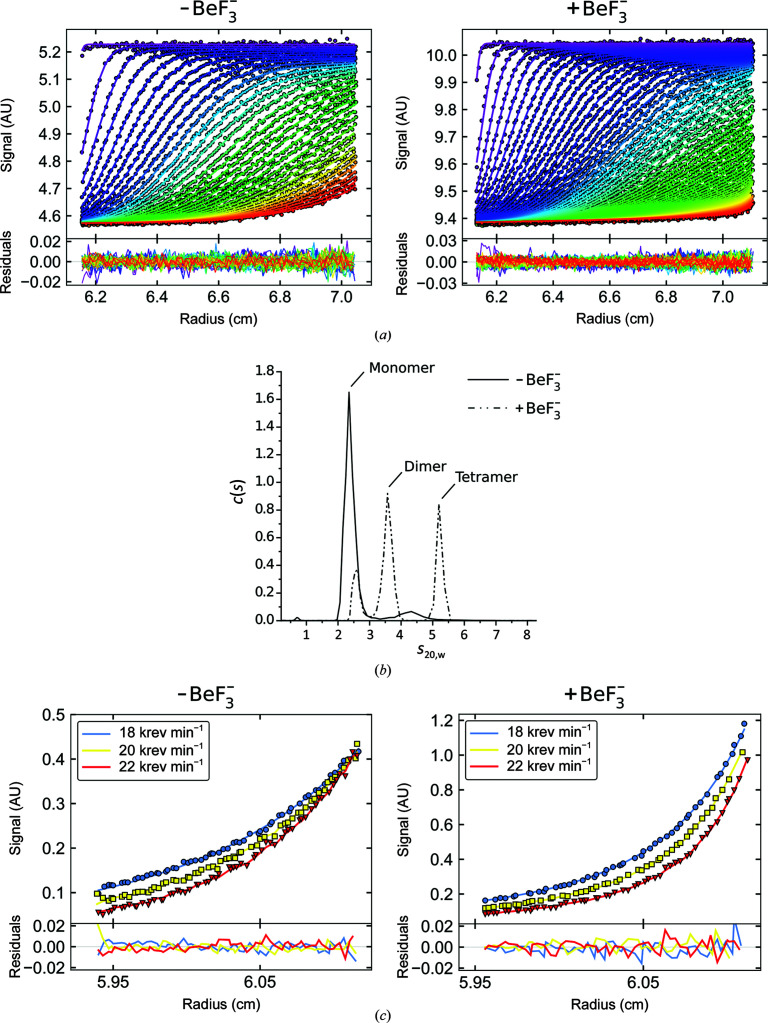
VanR_Sc_ oligomerizes in the presence of beryllium fluoride. (*a*) Sedimentation-velocity analytical ultracentrifugation. Experimental data are shown as circles and fits of the Lamm equation are shown as lines; residuals from the fit are shown below the data panels. Only every third boundary and third data point are shown for clarity. Measurements were performed at a 36.5 µ*M* monomer concentration at 20°C. (*b*) *c*(*s*) distributions derived from the fitting of the Lamm equation to the data shown in (*a*), as implemented in *SEDFIT*. The overall r.m.s.d. is 0.005 Å for both fits. This analysis shows evidence of monomer plus small amounts of a larger species in the absence of 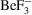 , and monomers, dimers and tetramers in the presence of 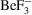 . These observations are consistent with the association constants derived from direct fitting of the sedimentation-velocity data to association models (see Supplementary Fig. S3 and Supplementary Table S3). (*c*) Sedimentation-equilibrium analytical ultracentrifugation. Representative data for 16.7 µ*M* protein in the absence of 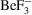 (left) and for 47.3 µ*M* protein in the presence of 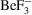 (right) are shown. Model fits are shown as lines for each of three radial absorbance boundaries collected at three speeds (18 000, 20 000 and 22 000 rev min^−1^); residuals for the model fitting are shown below the data panels. Data collected in the absence of 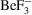 are best described by a weak monomer–dimer equilibrium model consistent with the two species observed by sedimentation velocity; data collected in the presence of 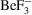 are best described by a monomer–dimer–tetramer equilibrium. Fit parameters are shown in Supplementary Table S4. Figures were prepared using *GUSSI*.

To test whether dimerization accompanies VanR_Sc_ phosphorylation in solution, we used analytical ultracentrifugation to probe the oligomerization state of the protein in the presence and absence of beryllium fluoride. In the absence of 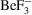 , sedimentation-velocity analysis revealed that the protein is largely present as a monomer, with small amounts of a more rapidly sedimenting species that might correspond to a compact dimer (Figs. 3 ▸ *a* and 3 ▸ *b*). Upon the addition of 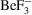 the profile shifts toward higher molecular-weight species, including a dimer and a putative tetramer. The polydispersity of the sample also increases in the presence of 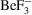 , with a particular increase in more extended species (Supplementary Fig. S4). We also examined the behavior of the protein in a sedimentation-equilibrium analysis; again, the addition of 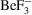 was accompanied by a clear shift toward higher-order species (Fig. 3 ▸ *c*).

A variety of models were used to fit the centrifugation data. For the sedimentation-velocity experiments performed in the absence of 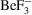 , the data were best fitted by a very weak monomer–dimer equilibrium model, with a *K*_d_ of >1 m*M* (Supplementary Table S3). The sedimentation-equilibrium data measured in the absence of 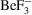 are also well described by a weak monomer–dimer equilibrium model, consistent with the sedimentation-velocity results; however, a somewhat lower estimate of *K*_d_ = 37 µ*M* was obtained (Supplementary Table S4). This may reflect differences in experimental conditions: the equilibrium experiments were performed at 4°C, while the velocity experiments were conducted at 20°C, and this temperature dependence might reflect a contribution from hydrophobic interactions during dimerization. In any case, however, it is evident that the monomer is the predominant species for the unphosphorylated protein.

In the presence of 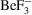 , the velocity data are well described by a monomer–dimer–tetramer equilibrium model, consistent with the multiple species observed in the *c*(*s*) distribution; estimated equilibrium dissociation constants for the two equilibria fall in the mid-micromolar range (Supplementary Table S3). The equilibrium data could also be fitted by a monomer–dimer–tetramer equilibrium model, providing a significantly better match than a monomer–dimer model. The dissociation constants for the equilibrium data were somewhat lower than those derived from the velocity data, as was seen for the data measured in the absence of 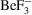 (Supplementary Table S4). However, both methods agree that the addition of 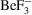 is accompanied by a distinct shift from monomer to dimers and higher-order species.

### 3.5. Comparison of the inactive and activated structures   

**Figure 4:**
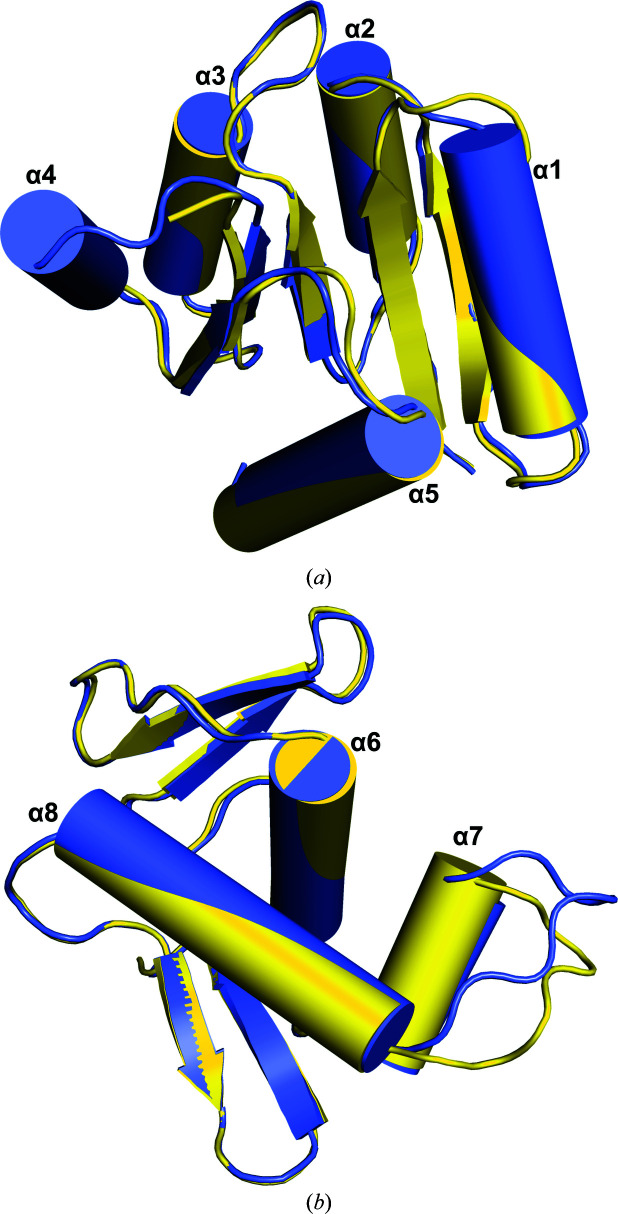
Domain comparison between the inactive and activated VanR_Sc_ structures. Inactive VanR_Sc_ is shown in yellow and activated VanR_Sc_ is shown in slate blue. (*a*) Superposition of the receiver domains; the largest differences are the lack of α4 in the inactive state and changes in conformation of the β4–α4 loop. (*b*) Superposition of the DNA-binding domains; the only significant difference between these two structures is the conformation of the α7–α8 loop.

The receiver domains of VanR_Sc_ in the inactive and activated states are extremely similar to each other, with an r.m.s.d. of 0.67 Å for all C^α^ atoms (Fig. 4 ▸ *a*). The most noticeable difference between the two receiver domains is the absence of helix α4 in the inactive structure, along with significantly different conformations of the loop connecting β4 to α4. In the inactive structure no electron density was observed for the entirety of α4, even though density is seen for the two flanking loops. To our knowledge, this level of α4 disorder has not been seen previously; however, this helix does adopt a range of different conformations in other response regulators, suggesting that conformational changes in α4 help to regulate activation (Buckler *et al.*, 2002 ▸; Bachhawat *et al.*, 2005 ▸; King-Scott *et al.*, 2007 ▸; Choudhury & Beis, 2013 ▸; see also PDB entry 3c97).

**Figure 5:**
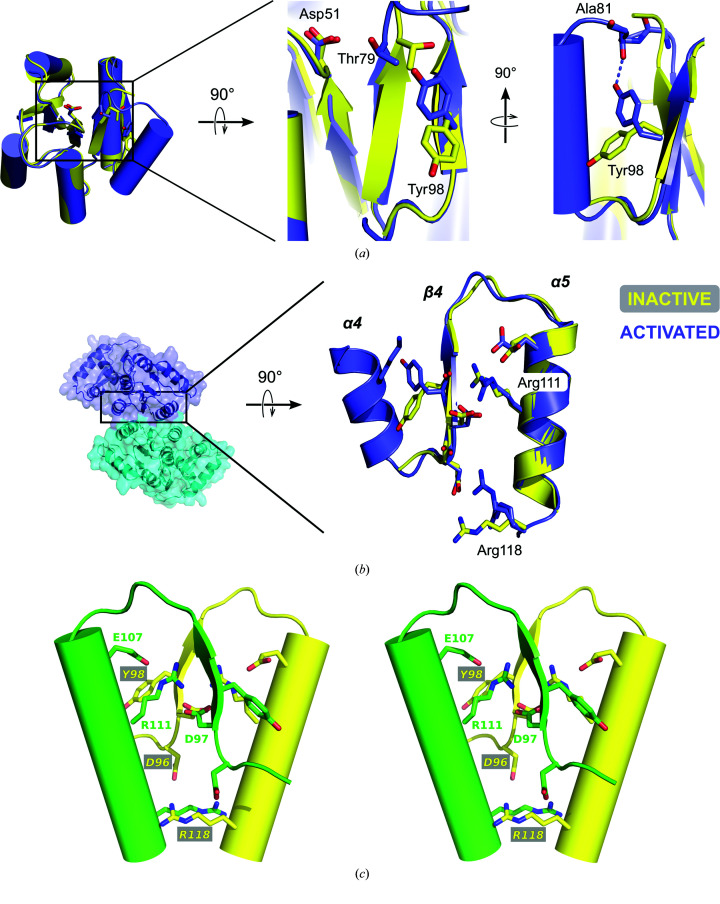
Structural changes associated with activation. (*a*) Changes in the switch residues. Inactive VanR_Sc_ is shown in yellow and activated VanR_Sc_ is shown in slate blue. (*b*) Superposition of α4–β5–α5 in the inactive and activated states highlights the side-chain movements of polar interface residues, notably Arg111 and Arg118. (*c*) Stereoview of a mock dimer interface produced by superposing the inactive structure onto the activated structure. The resulting two inactive-state protomers are shown in green and yellow. Side-chain conformations in the inactive state are incompatible with the activated-state dimer interface: Arg111 and Tyr98 are too far apart to interact, as are Arg118 and Asp96. Additionally, the inactive-state conformation of Arg118 would clash with its symmetry mate.

In addition to the gross changes surrounding α4, the two activity states exhibit smaller structural differences at the level of individual residues. One such difference is seen in the conserved amino-acid pair Thr79 and Tyr98. These correspond to the so-called switch residues, which favor different conformations in inactive versus activated receiver-domain structures (Gao *et al.*, 2019 ▸). Phosphorylation of Asp51 drives the conformational equilibrium towards a structure in which the side chain of Thr79 has moved towards the active site and formed a hydrogen bond with an O atom of the phosphoryl group. At the same time, Tyr98 rotates its side chain upwards to fill the space that had been occupied by Thr79 (Fig. 5 ▸ *a*). In this activated conformer, Tyr98 is stabilized by hydrogen bonding to the backbone carbonyl of Ala81 located in the β4–α4 loop. It is not clear which inactive-state interactions, if any, are disrupted by this movement of Tyr98, since the lack of density for α4 prevents potential interactors from being identified. However, it is clear that in the inactive state α4 cannot occupy the same position that it does in the activated state, because the side chain of Tyr98 would clash with the helical backbone, as shown in Fig. 5 ▸(*b*).

VanR_Sc_ does not form a dimer in the inactive state, and we reasoned that the transition from inactive monomer to activated dimer is likely to involve rearrangements of residues that create the α4–β5–α5 dimer interface. After superposition of the inactive and activated structures, we analyzed the positions of the residues that form the interface. When going from the inactive to the activated state, the side chains of Arg111 and Arg118 rotate in order to interact with the backbone carbonyls of Tyr98 and Asp96, respectively. If Arg118 did not move in this way, it would clash with its symmetry mate in the other half of the dimer (Fig. 5 ▸ *c*). Residues on α4 that contribute to the dimer interface (Ala88, Phe91 and Lys87) must also move in the shift from the inactive to the activated conformation, but the precise nature of these motions remains unknown, since α4 is disordered in the inactive state. However, it is likely that the interactions made by these three residues serve to stabilize α4 in its disorder-to-order transition.

Since activation of the response regulator promotes binding of its effector domain to its DNA target, we next compared the DNA-binding domains of VanR_Sc_ in the inactive and activated states. The conformations of the DNA-binding domains are similar in these two activity states, with an r.m.s.d. of 0.96 Å for all C^α^ atoms (Fig. 4 ▸ *b*). The most significant difference between the two conformations is the position of the α7–α8 loop. The conformation of this loop varies substantially in different response-regulator structures, which may reflect an inherent flexibility that allows optimal fitting at the protein–DNA interface (Robinson *et al.*, 2003 ▸).

### 3.6. Comparison of activated VanR_Sc_ with other activated OmpR/PhoB response regulators   

**Figure 6:**
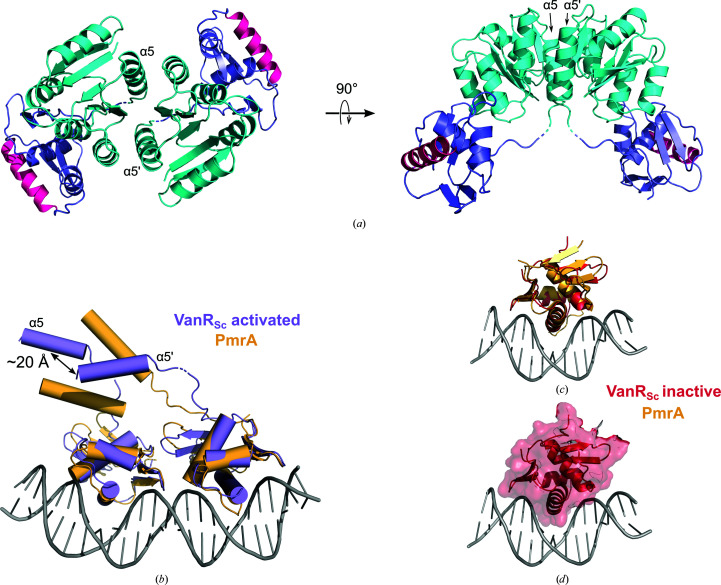
Modeling VanR_Sc_ assembly on DNA. (*a*) The activated VanR_Sc_ dimer shown in two orthogonal views. The receiver domain is colored cyan, the DNA-binding domain is colored slate blue and the recognition helix α8 is colored hot pink. The positions of the two symmetry-related copies of helix α5 are indicated. (*b*) To determine whether a head-to-tail configuration was consistent with the structure of activated VanR_Sc_, the VanR_Sc_ protomer (purple) was superposed upon each protomer of DNA-bound PmrA (light orange) by aligning the DNA-binding domains. For clarity, the only structural elements shown from the receiver domain are the α5 helices. (*c*, *d*) The DNA-binding domain in inactive VanR_Sc_ adopts a conformation that is consistent with DNA binding. The DNA-binding domain from inactive VanR_Sc_ (red) was superposed upon the corresponding domain from DNA-bound PmrA (light orange). (*c*) shows this superposition, while (*d*) shows a surface representation of VanR_Sc_. Both panels reveal that in this pose the VanR_Sc_ DNA-binding domain does not clash with the DNA. The PmrA structure was taken from PDB entry 4s04.

Many activated OmpR/PhoB response regulators (for example, KdpE and PmrA) assemble onto DNA with their DNA-binding domains arranged in a head-to-tail manner (Narayanan *et al.*, 2014 ▸; Lou *et al.*, 2015 ▸). Others (for example, OmpR) are able to bind in either a head-to-tail or a head-to-head orientation depending upon the specific sequences of the recognition sites (Maris *et al.*, 2005 ▸; Rhee *et al.*, 2008 ▸). However, in the dimer of activated VanR_Sc_ the two DNA-binding domains are positioned far from each other and adopt neither a head-to-tail nor a head-to-head orientation (Fig. 6 ▸ *a*). This difference in the positioning of the DNA-binding domains does not result from differences in the receiver domain, since KdpE and PmrA both form α4–β5–α5 dimers that are similar to the VanR_Sc_ dimer. Presumably, therefore, in the presence of DNA, the DNA-binding domain of VanR_Sc_ rearrange themselves so as to assemble head to tail. Such a rearrangement seems plausible, given the long linker connecting the receiver and DNA-binding domains of VanR_Sc_. This linker is approximately 35 Å in length, which is substantially longer than the linkers in KdpE and PmrA (19 and 30 Å, respectively). To test the plausibility of such a rearrangement, the DNA-binding domains of two copies of activated VanR_Sc_ were superposed on each of the two corresponding domains of the DNA-bound PmrA structure. Placing the two VanR_Sc_ monomers into this head-to-tail arrangement resulted in their α5 helices being positioned roughly parallel to one another, with the helix axes offset by approximately 20 Å (Fig. 6 ▸ *b*). The relative positions of these two helices are close to what is seen in the actual receiver-domain dimer, where these two helices are also roughly parallel to one another, with their axes separated by ∼14 Å at one end of the helices and ∼22 Å at the other (Fig. 6 ▸ *a*). Therefore, small adjustments in the flexible linkers should be sufficient to allow the activated VanR_Sc_ protein to form the canonical receiver-domain dimer, while at the same time allowing its DNA-binding domains to bind the target DNA in the expected head-to-tail conformation. We must note, however, that the precise DNA sequences recognized by VanR_Sc_ are not yet known: while the corresponding recognition sequences are known for the VanR proteins from type A and type B VRE, these proteins share less than 20% sequence identity with VanR_Sc_, and the upstream regions containing VanR sites are similarly divergent. Thus, in the absence of detailed knowledge about the recognition sites of VanR_Sc_, the relative positioning of the two protomers when bound to DNA remains a point of speculation.

### 3.7. Comparison of inactive VanR_Sc_ with other inactive OmpR/PhoB response regulators   

**Figure 7:**
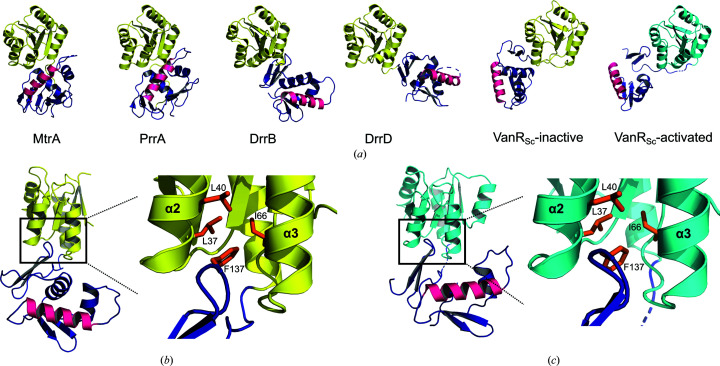
Modes of inactivation for OmpR/PhoB response regulators. (*a*) Inactive conformations of four different response regulators are shown, compared with the inactive conformation of VanR_Sc_. For the inactive response regulators, the receiver domains are shown in yellow, the DNA-binding domains in dark blue and the recognition helix α8 in hot pink. The activated VanR_Sc_ structure is also shown for the sake of comparison, with the receiver domain colored cyan. This panel was inspired by a figure in Friedland *et al.* (2007 ▸). PDB codes are as follows: MtrA, 2gwr; PrrA, 1ys6; DrrB, 1p2f; DrrD, 1kgs. (*b*) In the inactive VanR_Sc_ structure, the interdomain interaction involves the insertion of the β6–β7 turn from the DNA-binding domain into a cleft between helices α2 and α3 of the receiver domain, placing Phe137 into a hydrophobic pocket formed by Leu37, Leu40 and Ile66. (*c*) In the activated VanR_Sc_ structure a similar interdomain interaction is also formed, despite a change in the relative orientations of the two domains.

Why is unphosphorylated VanR_Sc_ inactive? The DNA-binding domain changes very little between the inactive and activated states, and even in the inactive state it adopts a conformation that appears competent to bind DNA (Figs. 6 ▸ *c* and 6 ▸ *d*). To address the molecular basis for inactivation, it is useful to consider inactive-state structures of other OmpR/PhoB-family response regulators. These structures suggest that more than one regulatory mechanism exists. For example, in PrrA and MtrA the DNA-binding domain is attached to the receiver domain so as to occlude the recognition helix, thereby preventing DNA binding (Fig. 7 ▸ *a*; Nowak *et al.*, 2006 ▸; Friedland *et al.*, 2007 ▸). In contrast, in DrrD and DrrB the recognition helix is not occluded in the inactive state; however, the two domains interact in a way that is thought to limit the mobility of the DNA-binding domain and thus hinder DNA binding (Buckler *et al.*, 2002 ▸; Robinson *et al.*, 2003 ▸). Finally, in addition to the inactivation mechanisms suggested by the structures described above, an additional possible mechanism is suggested by the observation that linker length and composition can alter response-regulator function (Mattison *et al.*, 2002 ▸; Walthers *et al.*, 2003 ▸); such linker effects may manifest dynamically, altering the relative mobility of the receiver and DNA-binding domains, and as such may prove difficult to capture structurally.

In the inactive VanR_Sc_ structure, the receiver and DNA-binding domains also interact via a small interdomain interface that is formed by the insertion of a β-turn from the DNA-binding domain into a cleft in the receiver domain, in an interaction that buries 260 Å^2^ of surface area (Fig. 7 ▸ *b*). This places the side chain of Phe137 into a hydrophobic pocket between helices α2 and α3, lined by the side chains of Leu37, Leu40 and Ile66. This interdomain interface is distinct from that seen in the PrrA and MtrA structures and does not occlude the recognition helix (Fig. 7 ▸ *a*). Interestingly, a similar interdomain interaction occurs in the structure of activated VanR_Sc_, despite the fact that the domains change their relative orientations in the inactive versus activated structures (Fig. 7 ▸ *c*). Given that this interdomain interaction appears to be able to accommodate some conformational variation, we suggest that a DNA-binding domain from one dimer might be able to interact intermolecularly with the receiver domain from another dimer, providing a potential explanation for the putative tetrameric species seen in the ultracentrifugation experiments. Even if this conjecture is correct, however, the biological relevance of oligomers larger than dimers remains unclear.

If the interdomain interaction does block activation by immobilizing the DNA-binding domain, as has been suggested for DrrD and DrrB, the interaction must be substantially stronger in the inactive state than in the activated state. This appears unlikely, given the similarity between the interdomain interfaces in the two states. Hence, we speculate that this interaction is sufficiently weak that it can be readily disrupted in the presence of the DNA target, freeing the DNA-binding domains so they may optimally orient themselves on the target. In conclusion, the structural evidence suggests that neither occlusion of the recognition helix nor immobilization of the DNA-binding domain is responsible for maintaining VanR_Sc_ in an inactive state.

In the structure of inactive VanR_Sc_, the linker connecting the receiver and DNA-binding domains is 27 Å in length, which presumably allows the DNA-binding domain to sample many different positions and orientations. This, together with the accessibility of the recognition helix, suggests that inactive VanR_Sc_ should be able to bind DNA. In support of this notion, the DNA-binding domain adopts a conformation that is compatible with target binding; for example, it can be superposed onto the DNA-bound structure of PmrA without clashing with the DNA (Figs. 6 ▸ *c* and 6 ▸ *d*). Indeed, the VanR orthologs from A- and B-type VRE have been shown to bind DNA in their unphosphorylated states, albeit much more weakly than the phosphorylated proteins (Holman *et al.*, 1994 ▸; Depardieu *et al.*, 2005 ▸). Therefore, we suggest that no structural impediment prevents VanR_Sc_ from binding DNA in its inactive state; however, this binding will be weak in the absence of dimerization. Once the protein is phosphorylated, the dimerization induced by the activating signal will enhance DNA binding through an avidity effect.

## 4. Conclusions   

We present here the first full-length OmpR/PhoB response regulator to be crystallized in both the inactive and activated states. The main structural differences between the two activity states center around the stability of α4 and the oligomeric state of the protein. Upon phosphorylation of Asp51, α4 transitions from a disordered to an ordered state, stabilizing key residues involved in forming the α4–β5–α5 dimer interface. We propose that this phosphorylation-induced dimerization provides an avidity effect that enhances DNA binding and promotes transcription. Overall, these structures suggest that the key feature required for the activation of transcription by VanR_Sc_ is its dimerization.

## Supplementary Material

PDB reference: VanR_Sc_, inactive, 7lz9

PDB reference: activated, 7lza

Supplementary Figures and Tables. DOI: 10.1107/S2059798321006288/ni5011sup1.pdf

Raw diffraction images for inactive VanRSc structure.: https://dx.doi.org/10.5281/zenodo.4594513

Raw diffraction images for activated VanRSc structure.: https://dx.doi.org/10.5281/zenodo.4593691

## Figures and Tables

**Table 1:** Data-collection and refinement statistics Values in parentheses are for the highest resolution shell.

	Inactive (PDB entry 7lz9)	Activated (PDB entry 7lza)
Data-collection statistics		
Diffraction source	Beamline 17-ID-1 (AMX), NSLS-II	Beamline 17-ID-1 (AMX), NSLS-II
Wavelength (Å)	0.920089	0.920091
Temperature (K)	100	100
Detector	EIGER 9M	EIGER 9M
Resolution range (Å)	32.75–2.30 (2.38–2.30)	28.85–2.03 (2.10–2.03)
Space group	*P*6_5_22	*P*6_5_22
*a*, *b*, *c* (Å)	74.38, 74.38, 138.23	97.37, 97.37, 118.65
α, β, γ (°)	90.0, 90.0, 120.0	90.0, 90.0, 120.0
Total No. of observations	27079 (24618)	94478 (8396)
No. of unique reflections	10592 (1033)	21574 (1981)
Average multiplicity	25.6 (23.8)	4.4 (4.2)
Completeness (%)	99.5 (98.9)	97.5 (91.8)
Mean *I*/σ(*I*)	10.9 (1.9)	15.3 (3.5)
Estimated Wilson *B* factor (Å^2^)	33.8	42.4
*R*_merge_ †	0.246 (1.953)	0.052 (0.406)
*R*_meas_ ‡	0.251 (1.996)	0.059 (0.463)
*R*_p.i.m._ §	0.049 (0.404)	0.028 (0.145)
CC_1/2_ ¶	0.998 (0.696)	0.998 (0.916)
Refinement and model statistics		
Resolution range (Å)	32.75–2.30 (2.38–2.30)	28.85–2.03 (2.10–2.03)
No. of reflections used	10062 (1033)	21562 (1978)
Reflections used for *R*_free_	529 (52)	1077 (98)
*R*_work_	0.202 (0.268)	0.182 (0.198)
*R*_free_	0.256 (0.339)	0.222 (0.252)
Solvent content (%)	44	62
No. of non-H atoms		
Protein	1594	1685
Water	28	184
Mg^2+^	1	1
{\rm BeF}_{3}^{-}	—	1
Average *B* value (Å^2^)	43.0	41.4
R.m.s. deviations from ideality		
Bond lengths (Å)	0.003	0.01
Angles (°)	0.59	0.85
Residue distribution in Ramachandran plot		
Most favored region (%)	97.6	97.2
Allowed (%)	2.4	2.8
Outliers (%)	0.0	0.0
Clashscore	1.86	3.52

† *R*_merge_ = \textstyle \sum_{hkl}\sum_{i}|I_{i}(hkl)- \langle I(hkl)\rangle|/\textstyle \sum_{hkl}\sum_{i}I_{i}(hkl), where *I_i_* (*hkl*) is the *i*th measurement of reflection *hkl*.

‡ *R*_meas_ (or redundancy-independent *R*_merge_) = \textstyle \sum_{hkl}\{N(hkl)/[N(hkl)-1]\}^{1/2}\sum_{i}|I_{i}(hkl)- \langle I(hkl)\rangle|/\textstyle \sum_{hkl}\sum_{i}I_{i}(hkl), where *I*_i_(*hkl*) is the *i*th measurement and *N*(*hkl*) is the redundancy of each unique reflection *hkl* (Diederichs & Karplus, 1997 ▸).

§ *R*_p.i.m._ = \textstyle \sum_{hkl}\{1/[N(hkl)-1]\}^{1/2}\sum_{i}|I_{i}(hkl)- \langle I(hkl)\rangle|/\textstyle \sum_{hkl}\sum_{i}I_{i}(hkl), where *I_*i*_* (*hkl*) is the *i*th measurement and *N*(*hkl*) is the redundancy of each unique reflection *hkl* (Weiss, 2001 ▸).

¶ CC_1/2_ is the correlation coefficient between two randomly chosen half data sets (Karplus & Diederichs, 2012 ▸).
