# Supplementary material for: Structures of full-length VanR from Streptomyces coelicolor in both the inactive and activated states
Source: Acta Crystallogr D Struct Biol. 2021 Jul 29;77(Pt 8):1027–39. doi: 10.1107/S2059798321006288 (PMC8329863; doi:10.1107/S2059798321006288)
Supplement: Supplementary file 1 [file d-77-01027-sup1.pdf]

STRUCTURAL
BIOLOGY

Volume 77 (2021)

Supporting information for article:

Structures of full-length VanR from *Streptomyces coelicolor* in both the inactive and activated states

Lina J. Maciunas, Nadia Porter, Paula J. Lee, Kushol Gupta and Patrick J. Loll

a.

b.

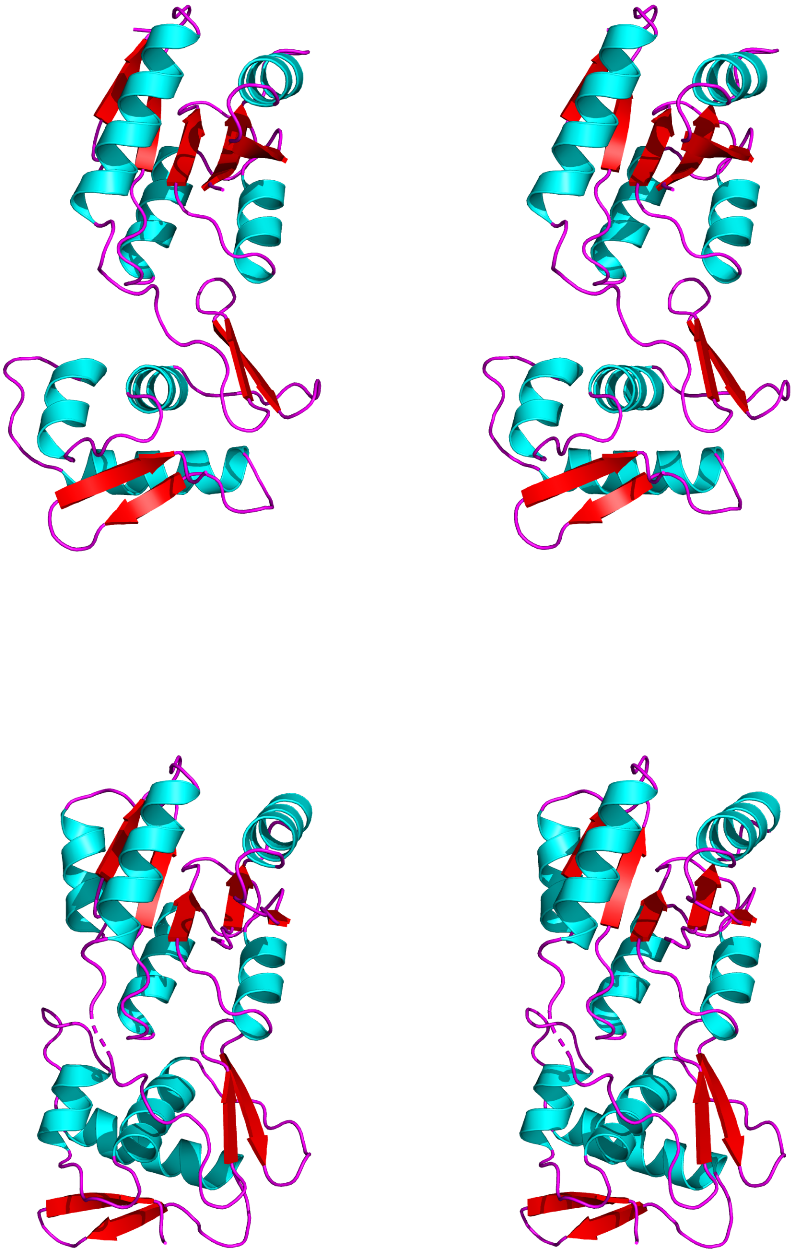

Figure S1. Stereo views of the structure of full-length $VanR_{Sc}$, showing both the inactive state (panel *a*) and the activated state (panel *b*). Color code is the same as that used in Figure 1.

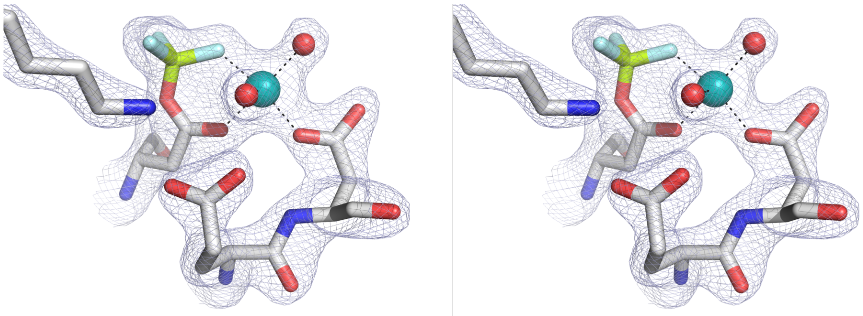

Figure S2. Stereo version of Fig. 2b, showing electron density associated with the phospho-mimetic bound to Asp-51.

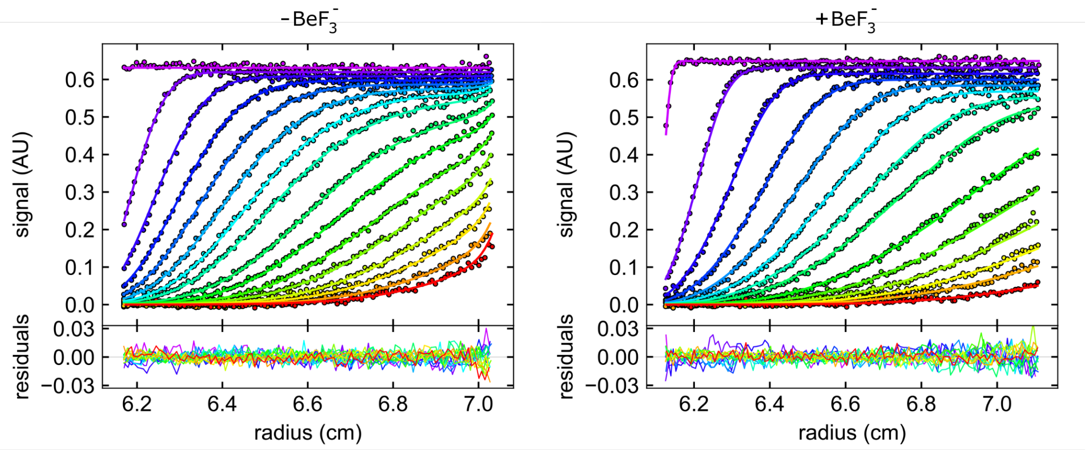

Monomer-dimer model

Monomer-dimer-tetramer model

Figure S3. *Association-model fitting of sedimentation-velocity ultracentrifugation data.* Only every third boundary and third datapoint are shown for clarity. Measurements were performed at 36.5 μM monomer concentrations at 20 °C. Data collected in the absence of BeF_3^- are well-described by a monomer-dimer equilibrium with a K_{d12} > 1 mM; a single-species monomer model also fits well (data not shown). Data collected in the presence of BeF_3^- are well-described by a monomer-dimer-tetramer equilibrium model, consistent with the species observed in the c(S) distributions. Model parameters are found in Table S3.

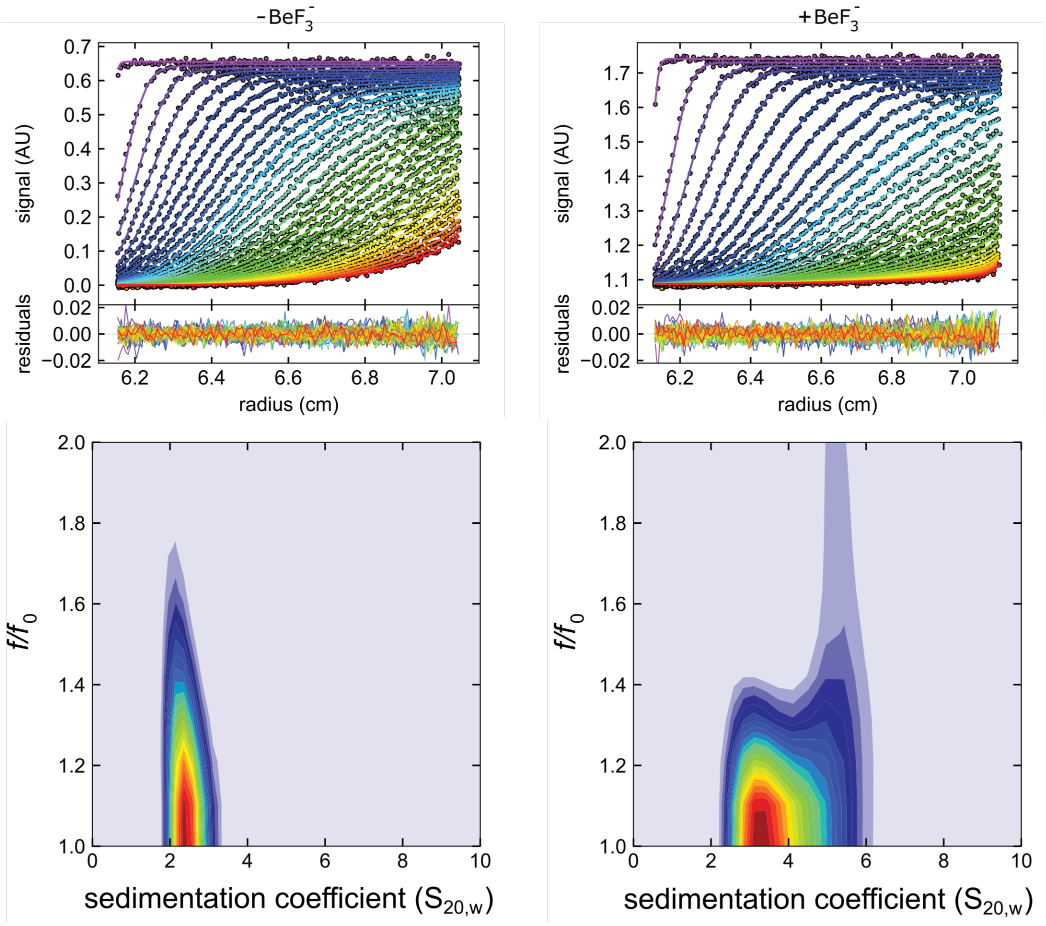

Figure S4. *c(S,f/f$_0$) analysis of sedimentation-velocity data*. Sedimentation data were analyzed using the c(S,f/f$_0$) model as implemented in Sedfit. The upper panels show fits of the experimental data (circles) to the model (lines), with residuals shown immediately below. Only every third boundary and third data point are shown for clarity. In the lower panels, 2-D shape and size distribution plots are shown, with sedimentation coefficient plotted along the *x*-axis and frictional ratio (f/f$_0$) plotted along the *y*-axis. Increasing values of f/f$_0$ correspond to highly elongated or non-globular species. The heat map indicates the species concentration, from lowest population density (blue) to highest (red). The addition of BeF_3^- is accompanied by a clear shift in size and shape, along with an increase in apparent polydispersity. S values were corrected to $S_{20,w}$.

Table S1: *Superposition of receiver domains*. The receiver domain of $VanR_{Sc}$ (residues 1-119) was superposed upon receiver domains of select OmpR/PhoB response regulators using the TM-align program (Zhang & Skolnick, 2005). RMSD values are for all Cα atoms contributing to the alignment.

			**$VanR_{Sc}$ (inactive)**		**$VanR_{Sc}$ (activated)**	
**PDB entry**	**Response regulator**	**Receiver domain residues**	**RMSD (Å)**	**Number of Cα atoms used in alignment:**	**RMSD (Å)**	**Number of Cα atoms used in alignment:**
1KGS	DrrD	1-121	1.33	101	1.74	110
1P2F	DrrB	1-118	1.50	102	1.51	114
4B09	BaeR	1-126	1.99	94	1.99	94
1YS6	PrrA	1-126	1.24	107	1.33	117
2GWR	MtrA	1-123	1.44	101	2.00	112
2OQR	RegX3	1-122	1.35	81	2.03	86
3R0J	PhoP	1-142	1.66	104	1.92	115
4KFC	KdpE	1-121	1.34	106	1.20	118
4S05	PmrA	1-119	1.25	107	1.12	119
1ZES	PhoB (activated)	1-122	1.21	106	1.11	118
1B00	PhoB (inactive)	1-123	1.46	107	1.54	116

Table S2: *Superposition of DNA-binding domains*. The DNA-binding domain of $VanR_{Sc}$ (residues 126-219) was superposed upon receiver domains of select OmpR/PhoB response regulators using TM-align. RMSD values are for all Cα atoms contributing to the alignment.

			**$VanR_{Sc}$ (inactive)**		**$VanR_{Sc}$ (activated)**	
**PDB entry**	**Response regulator**	**DNA-binding domain residues**	**RMSD (Å)**	**No. Cα atoms used in alignment**	**RMSD (Å)**	**No. Cα atoms used in alignment**
1KGS	DrrD	132 - 225	1.63	83	1.49	83
1P2F	DrrB	126 - 217	1.90	87	1.86	87
4B09	BaeR	141 - 240	1.93	84	2.23	86
1YS6	PrrA	140 - 233	1.49	90	1.96	90
2GWR	MtrA	134 - 228	1.40	88	1.66	88
2OQR	RegX3	137 - 226	1.49	84	1.74	84
3R0J	PhoP	157 - 246	1.36	87	1.28	87
4KFC	KdpE	134 - 225	1.95	88	2.04	87
4S05	PmrA	130 - 219	2.00	89	1.74	89
1QQI	PhoB - DNA	127-229	2.08	94	1.89	94
1GXP	PhoB + DNA	127-229	1.82	94	1.97	94
1OPC	OmpR	137-235	2.29	86	2.65	89

Table S3. Parameters Associated with Sedimentation-Velocity Analysis

Sample	Loaded Concentration (μM)	Model of Association	Mass (Daltons)	K_d (95% C.I.)	Global Reduced χ^2
$VanR_{Sc}$	34.6	Single Species (fixed)	24,947	*n.a.*	1.49
$VanR_{Sc}$	34.6	Two Species (fixed)	24,947 & 49,894	*n.a*	0.601
$VanR_{Sc}$	34.6	Monomer-Dimer	*n.a.*	>1 mM (n.d.)*	0.317
$VanR_{Sc}$	34.6	Monomer-Dimer-Tetramer	*n.a.*	K_{d12} = 1.01 mM (n.d.)<br>K_{d24} = 0.93 mM (n.d.)	1.03
$VanR_{Sc}$•BeF_3^-	34.6	Two Species (fixed)	24,947 & 49,894	*n.a.*	0.915
$VanR_{Sc}$•BeF_3^-	34.6	Three Species (fixed)	24,947, 49,894, & 99,788	*n.a.*	0.402
$VanR_{Sc}$•BeF_3^-	34.6	Monomer-Dimer-Tetramer	*n.a.*	K_{d12} = 40.1 μM (36.3 – 51.1)<br>K_{d24} = 23.9 μM (18.8 - 27.2)	0.393

* No CI could be determined for the monomer-dimer fit, so only an lower boundary for *K*d could be estimated.

Table S4. Parameters Associated with Sedimentation-Equilibrium Analysis

Sample	Loaded Concentrations (μM)	Speeds (Krpm)	Model of Association	Mass (Daltons)	K_d	Global Reduced χ^2
$VanR_{Sc}$	16.7, 25.4, 36.3, 46.7	18, 20, 22	Single Species (float)	31,188 ± 520	*n.a.*	1.94
$VanR_{Sc}$			Two Species (fixed)	24,947 & 49,894	*n.a*	1.14
$VanR_{Sc}$			Monomer-Dimer	*n.a.*	37 μM ± 0.6	1.88
$VanR_{Sc}$•BeF_3^-	16.2, 25.3, 38.7, 47.3	18, 20, 22	Single Species (float)	57,405 ± 764	*n.a.*	1.27
$VanR_{Sc}$•BeF_3^-			Single Species (fixed)	49,894	*n.a.*	1.49
$VanR_{Sc}$•BeF_3^-			Two Species (fixed)	24,947 & 49,894	*n.a.*	0.915
$VanR_{Sc}$•BeF_3^-			Three Species (fixed)	24,947, 49,894, & 99,788	*n.a.*	0.402
$VanR_{Sc}$•BeF_3^-			Monomer-Dimer	*n.a.*	*<<1 nM*	12.8
$VanR_{Sc}$•BeF_3^-			Monomer – Dimer - Tetramer	*n.a.*	K_{d12} *39.4* ± 23<br>K_{d24} *2.9* ± 1.5	0.388

Supplementary Information References

Zhang, Y. & Skolnick, J. (2005). *Nucleic Acids Res* **33**, 2302-2309.
